# The Function of the Spleen

**Published:** 1868-03

**Authors:** H. K. Jones

**Affiliations:** Jacksonville, Ill.


### CHICAGO MEDICAL EXAMINER.

N. S. DAVIS, M.D., EDITOR.

VOL. IX. MARCH, 1868. NO. 3.

### Original Contributions.

### ARTICLE VII.

### THE FUNCTION OF THE SPLEEN.

BY Dr. H. K. JONES, Jacksonville, Ill.

Read to the Morgan County Medical Society, January 9th, 1868.

It must, of course, be merely tedious to attempt to bring forward details of experiments, observations, and various views and doctrines found in medical literature, respecting the anatomy and physiology of viscus; and mere presumption to attempt conclusions, of matters which have proven a stumbling-stone to authorities of the greatest weight and eminence. But, therefore, the subject still lies at our door, challenging the scientific and philosophic scrutiny of this age, and to this end I would reproduce it to the attention of this Society.

In part, doubtless, owing to the inherent security of the function of this organ, and partly because of the demonstration of the practicability of existence without it, both in animals and in man; its importance in the animal economy, both as regards its function in health, and its pathological conditions and relations in the diseases of the body, is greatly underrated; and so our knowledge is abated and limited. For, if true, that its functions are vital and, therefore, its maladies absolutely and relatively grave, in that truth is to be found the stimulus to research, adequate to the erasure from medical literature of the opprobrium of this confessed superficialness and ignorance.

It must be fairly illustrative of the state of our knowledge or science of this subject, no longer ago than the pupilage of some of us who, in this Society, are but junior members, to quote in brief the views of so eminent an authority as Dr. Carpenter. He introduces the spleen as an organ having "no excretory duct, nor anything glandular in its structure." * * That, "in regard to the functions of the spleen, great uncertainty exists. That the spleen performs no action essential to life, has been repeatedly proved, by the experimental removal of it in many of the lower animals, and by the accidental loss of it in man, * * for after the immediate effects of the wound were recovered from, the vital functions were performed with no perceptible interruption, and health appeared to be completely restored." * * That "one of the numerous theories of its operation, which have at different times been brought forward, the one which is most satisfactory to the author, is that which regards it as a sort of *diverticulum*, or reservoir, which may serve to relieve the portal venous system from undue distension under a great variety of circumstances." * * That, "from the observations of Mr. Dobson, it appears that the spleen has its maximum volume at the time when the process of chymification is at an end, *viz.*, about five hours after food is taken; and that it is small and contains little blood, even hours later, when no food has been taken in the interval. Hence, he inferred that this organ is the receptacle for the increased quantity of blood which the system acquires from the food, and which cannot, without danger, be admitted into the bloodvessels generally, and that it regains its previous dimensions after the volume of the circulating fluid has been reduced by secretion."

Later, and contrariwise, Dr. Dalton propounds the case thus: "The spleen is a glandular organ, analogous in its minute structure to the solitary and aquinated glands of the small intestine, and to the lymphatic glands throughout the body. Like them, it is a gland without an excretory duct, and resembles, also, in this respect, the thyroid and thymus glands and the supra-renal capsules. All these organs have a structure which is evidently glandular in its nature, and yet the name of glands has been refused to them, because they have, as above mentioned, no duct, and produce, apparently, no distinct secretion. We have already seen, however, that a secretion may be produced in the interior of a glandular organ, like the sugar in the substance of the liver, and yet not be discharged by its excretory duct. The *veins* of the gland, in *this instance*, perform the part of excretory ducts. * * The action of such organs is, consequently, to modify the constitution of the blood. * * The precise alteration, however, which is effected in the blood during its passage through the splenic tissue has not yet been discovered."

The views of these eminent authorities in medical science and education, are here adduced for a double purpose:

1st. Of generalizing and fairly representing, as is supposed, the vagueness and contradictoriness of an accredited and standard literature of so modern a date; and,

2d. As affording us a convenient reduction of the matter of knowledge and to two recognized hypotheses, utterly inconsistent with each other, but within which—the one or the other—the truth may possibly be found, namely, the "*diverticulum*" or reservoir theory of Dr. Carpenter, on the one hand; and the *glandular* theory of Dr. Dalton, on the other hand.

Nor let it be supposed that Dr. Carpenter is the mere man of straw, set up for easy refutation, whilst, even at the present time, our prevailing splenic pathology and therapeutics, ideas of mere sanguineous plethora and engorgement, and consequent distension, hypertrophy, etc., are essentially grounded in the reservoir assumption.

This organ has a prominent position as a member and link in the golden circle of the great blood factories, and, accordingly, receives, in common with the stomach and liver, the first blood dispensed from the abdominal aorta. And, again, no other organ of the body receives so large a stream from the arterial fountains, in proportion to its size; and it returns the largest product in proportion to what it receives, its vein having six times the capacity of its artery. Its artery is larger than both the hepatic and gastric, and in its course dispenses twigs to the stomach and pancreas, as if its organ were in some special honor and dignity among its compeers. Again, it receives and acts upon only the purest blood of the body, and has no excretory duct—suggestive that its work can scarcely be subordinate or menial, neither depurating nor emunctory, but some superior service in the abdominal order and series looking primarily to a common end and use, namely, the initiation and consummation of the blood. And, accordingly, it transmits *all* its products through the sanguineous and lymphatic channels directly toward the fountain-head and palace and kingdom of the blood.

Thus, its position among the organs related to the production of the blood, the peculiar quantity of its arterial and venous streams, the absence of depurating and emunctory apparatus, the direction and destination of all its products, are facts whose concurring import amounts to a sort of *prima facie* evidence that this organ may be entitled to a distinguished rank as a primary blood generating factory.

But this is a mere outside view, to which the argument is by no means limited. Let us now look within, and see what we shall see. And here we will pass by its membranes, its large vessels, its trabecular meshes, etc.,—the framework only of the body, matters not in obscurity or dispute—and enter the intertrabecular spaces, the chambers and workshops of the factory, in which we are to see the peculiar substance of the spleen—at first view, a pulpy mass of "dark, reddish-brown" color, including corpuscles, capillary meshes, etc., altogether constituting the chief part of the entire bulk of this body.

Now, what have we here in this mass of confusion? Is it inexplicable chaos? Or shall rational, inductive science yet illustrate and demonstrate herein the most complete order and the most beauteous and productive skill?

The foundations of the answer to this very practical question were begun to be laid two centuries ago, when the Italian, Malpighi, the father of microscopic anatomy, first entered these hitherto dark chambers. Up to his time, the prevalent views among scientific men of Europe, in common with the earlier Greeks also, were only vague notions, some of which have still found their way down to our own times and country. Such as it was a useless and superfluous part of the body, an error of nature; or, that it was for the preservation of the equilibrium of the body; or, that it attracts watery materials from the stomach; or, that it was the sewer of the humor called atra bilis; or, that it fermented a menstruum for the stomach in digestion; or that it was the origin of laughter; or that it prepares the mucus to be secreted by the glands of the joints, etc.; and by rather more recent German and English authorities, (Schelnammer, Liston, Purcell, etc.) that it was a *diverticulum* for the blood in the more violent motions, etc., etc.

But Malpighi taught: "In the interstitial spaces of the spleen, we observe clusters of glands, or, rather, of vesicles or sacculi, in immense numbers, dispersed throughout the organ, and closely resembling bunches of grapes. These minute glands are of an oval figure, and about the size of the glands of the kidneys. I have always found them of a white color, and although the bloodvessels of the spleen may be fully injected with ink, and play around the glands, yet the latter still preserve their whiteness. They appear to consist of a membranous, but soft and very friable substance, the cavity in which is invisible by reason of its smallness, but still may be conjectured to exist, because these bodies collapse when cut. They are extremely numerous, indeed, almost innumerable, and are placed in a singular manner in the cells already described, all over the spleen, and hang from the productions of the capsule, or from fibres arising therefrom; consequently, from the extremities of the arteries and from the ends of the nerves, moreover, the ends of the arteries wind about them like tendrils of ivy. They usually hang in bunches, each cluster consisting of seven or eight glands."

Again, Winslow, the Danish anatomist, the disciple and later cotemporary, affirms: "The arteries, veins, and nerves, after entering the spleen, divide and subdivide into a great number of ramifications, and accompany each other to their very last divisions. The capillary extremities of all these ramifications, both arterial and venous, end in the small downy cells already described. Malpighi regarded these cells as distinct capsules or follicles, each containing a small gland. The extremities of the capillary ramifications seem to swim or float in the cells and to fill their downy tissue with blood. At the ends of several of these capillaries, I have observed small corpuscles disposed like bunches of grapes; and I have seen two little tubes going out of each corpuscle, one short and open, the other long and small, which latter was lost in the sides of the spleen," etc.

Other and later investigators have abundantly confirmed and amplified these discoveries into the further particulars, as, that these splenic bodies are not only covered on their exterior by a complete capillary network, but that they are also *penetrated* and permeated by this vascular distribution, and also that the entire splenic pulp is made up of several other varieties of glandular bodies, suggestive of complexity of function.

In brief, authority and opinion have gravitated to the general conclusion above stated from Dalton, namely, that this is a glandular organ, analogous to others of the body having no excretory duct, and in some way and extent concerned in modifying the constitution of the blood, but in what way may, perhaps, justly be said to have received no adequate scientific attempt until the very last years.

Ffteen or twenty years since, Virchow propounded and defended certain specific doctrines concerning the origin and morphologic changes of the blood: as, that "every permanent change which takes place in the condition of the circulating juices must be derived from definite points in the body, from individual organs or tissues. * * All the morphological elements of the blood, *whatever may be their nature*, are considered to be derived from sources external to the blood * * that in the ordinary course of things, the lymphatic glands and the spleen are really intermediately concerned in the production of the formed elements of the blood; and that, in particular, the *corpuscular constituents* of this fluid are really descendants of the cellular bodies of the lymphatic glands and the spleen, which have been set free in their interiors and conveyed into the current of the blood."

But it probably remains to our own time and countrymen to consummate the solution of this problem, and bear the torch-light of demonstrative science throughout its dark and most intricate mazes. And, already, sagacious and indefatigable explorers have reported important progress.

Within the last year, Prof. Salisbury, of Cleveland, Ohio, has published a paper which may justly be admitted to be the ablest contribution ever made to medical literature upon this subject; richest in original research, and in its facts and reasonings and pertinent suggestions. Besides the arterial terminations by capillary ramifications upon the Malpighian corpuscles, he distinguishes, also, terminations in glandular structures of two other varieties, namely:

1. In *tubular glandules* followed by simple capillaries; and,
2. In *tubular glandules* followed by oval splenic bodies.

In these, he discovers and traces the formation of fibrine cells, and their transformation into fibrinous cells. "These fibrin cells," says he, "during their slow, creeping passage through these thin-walled vessels, are gradually, after becoming fully mature, spun or developed into fibrine filaments of extreme tenuity and transparency, so that by the time they have reached the veins they enter them as fibrin filaments, or as cells far advanced in filamentous metamorphosis." Moreover, "there are two sets of these cells, an internal and an external—one arranged longitudinally, the other transversely. Both possess singular powers of contractility, like that of the muscular fibre," etc.

And again, "when the fibrin cells are developed into filaments, their nuclei are set at liberty, take up nerve and coloring matter, become highly transparent and homogenous, assume a flattened shape, and form *blood discs.* This process can be traced through all its stages in the living spleen." "The spleen and lacteal and lymphatic glands are the main organs that organize fibrin cells and blood discs." And, "of all these glands, the spleen is the most important and the most largely engaged in this cell-forming process."

Indulge me in the notice now, of one other fact claimed by this observer, namely: "In the spleen are found minute bodies containing a mesh of highly refractive cylindrical capillaries. * * They are always distended, highly refractive, and of a beautifully, pearly lustre. They resemble a meshwork of fine pearl-glass tubules. In these vessels may be noticed minute pearly drops, and on the membrane stretched across the meshes, the same kind of pearly drops aggregate into more or less spherical masses," and then, again, into larger globules, etc. And, finally: "These large globules take on a cell wall, and we have formed the genuine corpora amylacea." * * "They are the true myelin cells, or those cells which contain medullary nerve matter." And, again: "The contents of these cells after it has escaped from the cases, become aggregated into large globular masses, and from the sides of which are developed beautiful, double-walled, pearly, tubular filaments, precisely like those of *nerve tissue.*"

Now the above series of observations and facts, connected and corroborative, as landmarks indicating the progress of this inquiry through the last two centuries, are believed to embrace grounds and conclusions from which histological and physiological science will not recede, namely: Of this organ, its duct is not in the direction of depuration or emunctory work. It receives more arterial blood than can be appropriated to its own nutrition, and its large venous duct conveys away larger products than the residue and debris of its functions. And its products are sent exclusively towards the fountains. Also, its interstitial spaces are filled up with a variety of glandular bodies, unto which, as unto so many furnaces, and forges, and mills, are the ultimate ramifications and determinations of the apporting arterial streams, and from which bodies also arise the absorbent and capillary origins of the deporting venous and lymphatic trunks, which take up and bear away towards the sources of the blood materials unlike those received. So that, with the exception possibly of a minor system of anastomosing capillaries, the arterial stream, with all its vast supplies, must pass between the upper and the nether millstone of glandular structure, to be resolved into its principles and reorganized into new forms for the perpetual reproduction and reconstruction of the blood.

And, lastly, one stands amid these workshops and sees the actual initiaments and progressions, first, of the fibrin cell with its ultimate development into the contractile filament, looking to the end of muscular nutrition; and then its nucleus developed and formed into the red-blood globule; and this, again, is here ermined and crimsoned, and sent away freighted with treasures for distant ports of the realm. And here is witnessed also the generation of the myeline cell, with its medullary substance and nerve filament, looking to the end of the endowment of the blood-disc, the surrounding and protection of the axis cylinder of the nerve conduits, and the ultimate nutrition and reproduction of the essential substance of the brains and the nerves.

Finally, this view is amply corroborated in the phenomena of ascertained pathological states of this organ—as,

1. In the case of deficiencies of coloring matter in the blood, as also in diseases of discolorations of this fluid, confessedly phenomenal of congestions, irritations, inflammations, indurations, hypertrophies, etc., of this viscus. Virchow, after a scrutinizing survey of the subjects of leukæmia, melanæmia, melanotic tumors, etc., concludes "that all the facts with which we are acquainted, concerning these conditions, indicate that the contamination of the blood has its rise in a definite organ, and that this organ, as in the colorless blood corpuscles, is generally the spleen."

2. In the case of the removal of this gland from the body, the voracity and increased ferocity of the animal until the consequent deficiencies of the blood in the above-named materials for muscular and nervous nutrition are compensated by the vicarious functions of associated and kindred organs, afford additional corroboration.

3. Another interesting and important class of corroborative facts are the sensational, intellectual, and moral symptoms of the patient, in the functional disorders of this organ, commonly reputed, not only in popular, but also in professional parlance, to be splenetic affection of the mind, such as general irritability, particularly of the irascible dispositions, peevishness, moroseness, apprehensiveness, despondencies, anxieties, nostalgia, and, possibly, various monomanias, etc. This class of symptoms direct our attention, unmistakably, to deficiencies in the elements of cerebral and nervous nutriment, namely, medullary and nerve filament substances, the generation of which in the spleen has been ascertained as above mentioned. And, furthermore, Prof. Salisbury observes: "These (myeline cell substances) are taken up by the blood-discs which transmit them to their destinations. This matter communicates a high degree of plasticity and refractive pearly lustre to the discs. * * * The myeline performs the office of surrounding the axis cylinder of the nerves, forming an insulating tunic between it and the nerve sheath, which prevents the nerve from escaping in its transit, save at the terminus."

The relation of these matters to the nutrition and healthy and complete functions of the nervous system is so palpable, that the Professor insists "that by simply examining the blood microscopically, it can readily be determined whether a person is actively engaged in mental pursuits, under the influence of nervous exhaustion of any kind, or whether he is purely devoted to physical undertakings, is strong and robust, and is laboring under no influence which is exhausting his nerve tissue."

If now, in conclusion, it should be true, as the above chain of facts and reasonings tend to show, that in this organ as a *factory*—not as a mere *secreting* gland—are the generations and initiaments *de novo* of the fibrin cell and contractile filaments appertaining to the support of muscular tissue, and, also, of the myeline cell and nervous filament appertaining to the support of nervous nutrition and function; and, also, of the red blood corpuscle as, possibly, the carrier of certain supplies to glandular ports and marts abroad; and, finally, if on the other hand, there be found, concomitant with its abnormal conditions, muscular inanitions and debilities, mental and moral irritabilities and depressions, disturbance of the proportion of the red and white blood cells, discolorations of the blood, etc., etc., we are, then, already on pretty fair advantage ground for the ultimate realization of a truly scientific physiology and pathology of this viscus. And this event may also possibly be hailed as the signal of deliverance for the liver and stomach and other of its neighbors that have, through the long time, endured and suffered untold indignities and penal inflictions for offenses not their own.

